# The cost of chronicity: *Pseudomonas aeruginosa*’s silent evolution in the urinary tract

**DOI:** 10.1128/spectrum.03381-25

**Published:** 2025-11-25

**Authors:** Sébastien Bontemps-Gallo

**Affiliations:** 1 University of Lille, CNRS, Inserm, CHU Lille, Institut Pasteur de Lille, U1019 - UMR 9017 - CIIL - Center for Infection and Immunity of Lille 27023, Lille, France; Universidad Maimonides, Buenos Aires, Argentina

**Keywords:** *Pseudomonas aeruginosa*, stress adaptation, urinary tract infection, virulence, persistence, Koch postulate

## Abstract

More than a century after Koch’s postulates defined the microbial causes of disease, Stanley Falkow reminded us that pathogenicity is not an intrinsic property but a contextual outcome of microbial adaptation. The recent study by C. Martin-Duval, S. Dahyot, I. Coquisart, B. Bernay, et al. (Microbiol Spectr 13:e00456-25, 2025, https://doi.org/10.1128/spectrum.00456-25) illustrates this concept. By combining genomic, phenotypic, and proteomic analyses of sequential urinary isolates, the authors show how *Pseudomonas aeruginosa* progressively abandons virulence traits while reinforcing metabolic resilience during urinary tract infections. This commentary discusses how these findings challenge classical views of pathogenic success, highlighting the “cost of chronicity” as an adaptive trade-off between acute infection and long-term colonization. The urinary tract thus emerges not as a battlefield, but as a niche of persistence for *P. aeruginosa*.

## COMMENTARY

More than a century after Robert Koch proposed his postulates to demonstrate the causal relationship between a microorganism and a disease, Stanley Falkow reframed the debate (1, 2). For him, virulence is not an intrinsic trait of a bacterium but a dynamic relationship, i.e., the phenotypic expression of adaptive success. Bacteria do not evolve to cause disease, but to survive.

The study by Martin-Duval et al. in *Microbiology Spectrum* (3) provides a compelling modern illustration of this principle. Through genomic, phenotypic, and proteomic analyses of *Pseudomonas aeruginosa* urinary isolates collected sequentially from patients with recurrent infections, the authors show that chronic persistence arises not from enhanced virulence but from its progressive loss. The urinary tract thus becomes a model of Falkow’s paradox: the pathogen that survives is not the one that fights hardest, but the one that learns to live quietly.

*P. aeruginosa* is a paradigmatic opportunist. Usually harmless to healthy individuals, *P. aeruginosa* can become a pathogen in immunocompromised hosts. Its capacity to form biofilms and its resistance to antimicrobial agents make the treatment of this pathogen difficult, often leading to recurrent relapses and the establishment of chronic infections. Moreover, *P. aeruginosa*’s large genome, reflecting its ubiquitous nature, encodes remarkable metabolic adaptability, allowing it to thrive in the cystic fibrosis lung, in burn wounds, or within the urinary tract (4). Yet, unlike the respiratory niche, the urinary tract is metabolically, chemically dynamic but immunologically restrained.

Martin-Duval et al. compared early and late isolates from three patients experiencing recurrent urinary infections, two of whom were immunocompromised. Choosing patient-derived isolates rather than laboratory strains or experimental evolution models provides a biologically relevant framework for understanding the bacterium’s intra-patient adaptive capacities. At the genomic level, late isolates carried large deletions and numerous single-nucleotide polymorphisms affecting regulatory systems, motility, and metabolism. Despite patient-specific genomic changes, the phenotypic outcome was convergent: slower growth, decreased motility, attenuated virulence, and a shift toward energy-efficient metabolism.

Chronic persistence carries an evolutionary cost for the bacterium. *P. aeruginosa* trades virulence for persistence. Indeed, late isolates were less virulent when tested into a susceptible host model (*Galleria mellonella*). Those late isolates recovered after months in the patient, compared with the initial isolate, showed reduced expression of genes involved in flagellar assembly, chemotaxis, and the type III secretion system. Motility assays confirmed a near-complete loss of swimming ability in late isolates from two of the three patients. These features are essential for acute infection but prove dispensable for long-term colonization.

Proteomic analyses revealed enrichment of enzymes involved in the glyoxylate shunt, pyruvate metabolism, and oxidative phosphorylation. These pathways support efficient energy use under nutrient-limited conditions. Such metabolic remodeling reflects that described in chronic cystic fibrosis infections and underscores the metabolic optimization that supports persistence.

An unexpected observation was the disappearance of siderophore-related proteins (pyoverdine and pyochelin) in late isolates grown in human urine, despite its iron-limited nature. The downregulation of *pchD* and *sodM* transcripts confirmed this shift. This observation is counterintuitive since human urine is an iron-poor environment (5), suggesting that *P. aeruginosa* establishes long-term residence without prioritizing maximal resource acquisition.

A methodological strength of the study is the use of pooled human urine. Proteomic divergence between human urine and artificial urine medium cultures revealed that artificial formulations fail to capture the biochemical complexity of the urinary environment. Such findings have broad implications: *in vitro* mimicking media, even those approximating physiological conditions, may not reflect the full extent of bacterial metabolic adaptation. In human urine, *P. aeruginosa* modulated the expression of osmoprotectant transporters (OpuCA, OpuCD) and enzymes linked to osmotic stress tolerance, reflecting adaptation to the high osmolarity (up to 1,400 mOsm, compared with the physiological standard of about 300 mOsm in blood) and variable pH of urine. This observation of adaptive capacity highlights the continuum between pathogenicity and adaptation in a single bacterial species.

The evolutionary patterns described by Martin-Duval et al. echo those long observed in cystic fibrosis isolates: loss of motility, downregulation of acute virulence determinants, and remodeling of central metabolism. Across niches as distinct as the lung and the bladder, *P. aeruginosa* shows a convergence of persistence mechanisms. This finding suggests that the establishment of chronic infection may represent a reproducible persistence strategy. In other words, although the specific genes affected by deletion or downregulation differ, the main phenotypic traits that must be silenced, or at least reduced, to shift from acute infection to long-term persistence are the same, regardless of the colonized organ.

This study also highlights another paradox. From a genomic perspective, the large deletions might suggest that *P. aeruginosa* becomes “diminished” or “weakened.” Yet, this apparent loss confers an ecological advantage, enabling stable and lasting colonization of a new niche. Martin-Duval et al. also challenge Koch’s postulates by showing that bacterial pathogenicity is not a binary phenomenon: virulent or non-virulent. This perspective underscores that the “cost of chronicity” is both biological (genome reduction for the bacterium) and conceptual (how we define virulence itself) (2, 6). This biologically relevant example offers insight into the adaptive journey of a bacterial species oscillating between virulence, persistence, and saprophytism.

Beyond the lens of virulence, it is worth recalling that bacteria live not to cause disease but to grow and divide. The *P. aeruginosa* lineage adapted to chronic urinary infection illustrates this point. Persistence depends on the silencing of virulence determinants. In contrast to Falkow’s original view, it is the loss, not the presence, of virulence factors that sets the bacterium’s pathogenic trajectory, enabling its long-term establishment within the host.
